# Association between occupational stress and subclinical depression in Chinese primary healthcare workers

**DOI:** 10.3389/fpsyt.2023.1238603

**Published:** 2023-11-07

**Authors:** Jiao Zhang, Lingzhong Xu, Wenzhe Qin, Aijun Xu

**Affiliations:** ^1^School of Health Economics and Management, Nanjing University of Chinese Medicine, Nanjing, China; ^2^Jiangsu Research Center for Major Health Risk Management and TCM Control Policy, Nanjing University of Chinese Medicine, Nanjing, China; ^3^Centre for Health Management and Policy Research, School of Public Health, Cheeloo College of Medicine, Shandong University, Jinan, China; ^4^School of Nursing, Nanjing University of Chinese Medicine, Nanjing, China

**Keywords:** occupational stress, subclinical depression, primary healthcare worker, gender difference, challenge & hindrance stressors

## Abstract

**Background:**

Wellbeing of healthcare workers is crucial for the effective functioning of primary health systems. This study aimed to examine the association between occupational stress and subclinical depression among primary healthcare workers, and to establish the foundation for future preventive strategies.

**Methods:**

A cross-sectional study was conducted in Tai’an City, Shandong Province, China. Data were collected from 832 medical staff in primary health institutions using a structured self-administered questionnaire. The participants completed the Challenge and Hindrance-Related Self-Reported Stress (C-HSS) Scale and Patient Health Questionnaire-9. Multivariable logistic regression analysis was conducted to explore the relationship between occupational stress and subclinical depression among primary healthcare workers.

**Results:**

The prevalence of subclinical depression among primary healthcare workers was 11.66%. Participants with subclinical depression have a significant higher level of occupational stress (including challenge-stress and hindrance-stress). Regression analysis result indicated that higher level of occupational stress was significantly associated with more severe subclinical depression, and the risk of subclinical depression remained after adjusting other covariates (OR = 4.57, 95%CI, 3.14–6.63). The association between challenge-stress and subclinical depression was not statistically significant when controlling for hindrance-stress. Subgroup analysis showed that male healthcare workers who perceived higher level of challenge stress were more likely to develop subclinical depression than female healthcare workers.

**Conclusion:**

The level of subclinical depression among Chinese primary healthcare workers was high, and occupational stress especially hindrance stress may contribute to subclinical depression. Findings were also robust in subgroup analysis after adjusting for other covariates. These findings emphasize the importance of occupational stress psychosocial interventions to decrease the risk of developing depression among the primary healthcare workers.

## 1. Introduction

Occupational stress is generally defined as a harmful physical and emotional response to an individual’s physiological and/or psychological state in the work environment, which is very common among medical professionals (1, 2). Occupational stress occurs when worker’s working resources, abilities and needs are insufficient to cope with demands and pressure of work, resulting in individual change in the their physiological and/psychological or state (3). Occupational stress among primary healthcare workers is relatively high when compared to workers in other sectors. Previous study has reported that this is particularly true for general practitioners, a group that ranks highly among the healthcare professions in respect of occupational stress (4). The situation for Chinese primary healthcare workers is much worse, as they face the enormous workplace pressure. A survey conducted in southwestern China showed that 78.39% of primary health workers experienced occupational stress (5). In recent year, primary healthcare institutions in China have been experiencing medical workers shortages and high turnover. The possible contributor to these problems among the primary healthcare workforce is occupational stress (6). Occupational stress is associated with an increase in burnout, a decrease in mental health, job satisfaction and performance, which will subsequently affect the quality of the nation’s broader healthcare system.

Depression is a worldwide spread disease with significant impact on health and life quality. Available evidence has shown that medical staff have a higher prevalence of depression in comparison with general population (7–9). Results from a previous study in China indicated that the prevalence of depression among primary-care physicians was up to 31.7% (10). Primary healthcare workers are the gatekeepers of population health, because they are usually the first contact point between individuals and the health care system, and they are the key to prevent, monitor and manage various health problems. In addition, in the context of preventing and controlling the COVID-19 epidemic, primary healthcare workers on the frontlines undertake increasing responsibility and multitasking works, are more vulnerable to psychological deficits such as depression (11, 12). Evidence has shown that subclinical levels of depressive symptoms are associated with significant impairment as well as an increased risk for developing major depressive disorder (13). Early identification of subclinical depression in primary healthcare workers thus constitutes an important goal.

Previous research highlighted that occupational stress can affect depression symptoms among medical staff, and more support is needed for these vulnerable groups (14, 15). However, there is extremely few empirical studies that focused on the primary healthcare workers in the context of implementation of relevant policies on strengthening the construction of the primary healthcare system. Additionally, most previous research on occupational stress did not distinguish between challenge stress (work stress that facilities career development, such as shift positions, job responsibility, and workload) and hindrance stress (considered to be unbearable work pressure, such as heavy work policies, conflicts with others, and job insecurity), and whether there is difference in the effect of these stress on subclinical depression of primary healthcare workers remains to be determined. Therefore, in this paper, we aimed to investigated the association between occupational stress and subclinical depression among healthcare workers in Chinese primary healthcare settings. We also explored how sex and residence might moderate the above association to provide information for developing interventions that are tailored to specific situations.

## 2. Methods

### 2.1. Study population

Our data was collected from a filed survey conducted in Tai’an, China, in August 2020. Multi-stage random sampling method was adopted to select the representative sample of primary healthcare workers. First, three or four townships were randomly selected from each district (county) in Tai’an city, with a total of 20 township health centers (community health service centers); Secondly, 8 villages (communities) were randomly selected from each township, with a total of 160 village clinics (community health service stations); Thirdly, healthcare workers were enrolled from the above selected primary health institutions. The inclusion criteria were all primary healthcare workers on duty on the day of survey, including general practitioners, village doctors, nurses, public health workers and other members of family doctor contract service team. Written consent was collected after clearly explaining to respondents the survey data would be used. After a pilot survey of 10 healthcare workers, we made minor adjustments to the content and layout of the questionnaire to improve its rationality and acceptability. A centralized self-filling questionnaire approach was adopted in each primary health institutions and took an average of 15–30 min to complete. During the self-filling process, two research assistants introduced the study to every participant and provided guidance on any queries they might have and checked the quality of questionnaire completion. Finally, there were totally 832 usable, eligible responses after excluding 13 incomplete records.

### 2.2. Assessment of occupational stress

Occupational stress was measured using the 11-item Challenge and Hindrance-Related Self-Reported Stress (C-HSS) Scale. Each item was evaluated with five-point Likert scale (1 = no stress; 5 = great stress), with higher total scores reflecting greater levels of occupational stress (16). The C-HSS scale was proven to have high reliability (Cronbach α = 0.901) in this study, and Cronbach α for the challenge stress and hindrance stress scale was 0.923 and 0.857, respectively.

### 2.3. Assessment of subclinical depression

Subclinical depression was screened by using Patient health questionnaire-9 (PHQ-9), where each symptom item should be answered based on the past 2 weeks and scored according to the response options: 0 (not at all), 1 (few days), 2 (more than half the days), and 3 (almost every day). The total score ranged from 0 to 27, with a higher score denoting a higher level of subclinical depression. The PHQ-9 was the most widely used instrument for screening subclinical depression in primary health care (17, 18). A total score ≥ 10 was proven to be of more clinical significance in identifying depression symptoms (18, 19). Therefore, 10 points was taken as the cut-off point in present analysis. Cronbach’s α for the PHQ-9 in this study sample was 0.929.

### 2.4. Covariates

Sociodemographic variables included age (in years, ≤30, 31 ~ 40, 41 ~ 50, ≥51), sex (male, female), marital status (couple, single), residential region (rural, urban), education (in years, ≤12, 13 ~ 15, ≥16) and personal annual income (five income quintiles: quintile 1 (Q1) is the poorest and quintile 4 (Q4) is the richest). The occupational characteristics and chronic condition were also included as covariates in this study. Occupational characteristics includes professional title (none, primary, intermediate or higher) and work experience (in years, ≤10, 11 ~ 20, 21 ~ 30, ≥31); Chronic condition (yes, no) was defined as any reported been diagnosed with one or more chronic diseases.

### 2.5. Statistical analysis

Data were described using either numbers (proportion) or means (standard deviations, SD), where appropriate. We compared occupational stress between baseline characteristics by using t-test (for two categories) or one-way ANOVA (for three or more categories). Baseline characteristics according to the onset of subclinical depression were examined using Chi-squared tests. Further, we used multivariable logistic regression analysis to estimate the prospective association between occupational stress (challenge stress, hindrance stress) and subclinical depression. We also conducted subgroup analysis for sex groups (male and female) and residential region groups (rural and urban). Odds ratios (ORs) and the 95% confidence intervals (CIs) for subclinical depression were estimated in both crude and adjusted models. All data were analyzed using Stata 17.0. The *p*-value<0.05 was considered to be statistically significant.

## 3. Results

### 3.1. Characteristic of participants

A total of 832 primary healthcare workers were included in the analysis [mean (SD) age 42.9 (8.5) years], 53.37% of them were male and 46.63% were female. More than half of the participants (58.89%) lived in rural areas. The overall mean (SD) occupational stress score of participants was 3.19 (0.72). Those who were older, male, couple, living in rural region, having lower level of income, having more work years, and having chronic condition reported higher level of occupational stress than their counterparts (all *p* < 0.001) (see Table 1). The sample characteristics by subclinical depression are shown in Table 2. The prevalence of subclinical depression (i.e., those that reached our cut-off for subclinical depression) among primary healthcare workers was 11.66% (97/832). Participants with chronic condition were more likely to suffer from subclinical depression than their counterparts (*p* = 0.001).

**Table 1:** Sociodemographic characteristic and occupational stress of participants (*N* = 832).

Characteristic	No. (%)	Occupational stress (M ± SD)	t/F	*p*-value
Observations	832 (100.00)	3.19 ± 0.72		
Age, year			7.84	<0.001
≤30	62 (7.45)	2.80 ± 0.70		
31 ~ 40	241 (28.97)	3.18 ± 0.69		
41 ~ 50	392 (47.12)	3.22 ± 0.73		
≥51	137 (16.47)	3.31 ± 0.72		
Sex			6.90	<0.001
Male	444 (53.37)	3.35 ± 0.71		
Female	388 (46.63)	3.01 ± 0.70		
Marital status			−2.04	0.041
Single	71 (8.53)	3.03 ± 0.87		
Couple	761 (91.47)	3.21 ± 0.70		
Residential region			4.75	<0.001
Rural	490 (58.89)	3.29 ± 0.73		
Urban	342 (41.11)	3.05 ± 0.69		
Education, year			1.44	0.238
≤12	294 (35.34)	3.20 ± 0.74		
13 ~ 15	360 (43.27)	3.15 ± 0.71		
≥16	178 (21.39)	3.26 ± 0.71		
Income			3.30	0.011
Q1 (Lowest)	183 (22.00)	3.35 ± 0.76		
Q2	152 (18.27)	3.20 ± 0.73		
Q3	181 (21.75)	3.17 ± 0.71		
Q4	140 (16.83)	3.16 ± 0.81		
Q5 (Highest)	176 (21.15)	3.09 ± 0.58		
Professional title			1.00	0.370
None	321 (38.58)	3.23 ± 0.75		
Primary	353 (42.43)	3.15 ± 0.74		
Intermediate or higher	158 (18.99)	3.21 ± 0.62		
Work experience, year			9.73	<0.001
≤10	204 (24.52)	2.96 ± 0.74		
11 ~ 20	227 (27.28)	3.26 ± 0.68		
21 ~ 30	301 (36.18)	3.25 ± 0.72		
≥31	100 (12.02)	3.33 ± 0.69		
Chronic condition			4.31	<0.001
Yes	160 (19.23)	3.41 ± 0.71		
No	672 (80.77)	3.14 ± 0.71		

**Table 2:** Characteristic of participants by subclinical depression (*N* = 832).

Characteristic	Depression [*N* (%)]		χ^2^	*P*-value^a^
	Yes	No		
Observations	97 (11.66)	735 (88.34)		
Age, year			1.96	0.580
≤30	9 (14.52)	53 (85.48)		
31 ~ 40	31 (12.86)	210 (87.14)		
41 ~ 50	45 (11.48)	347 (88.52)		
≥51	12 (8.76)	125 (91.24)		
Sex			3.18	0.075
Male	60 (13.51)	384 (86.49)		
Female	37 (9.54)	351 (90.46)		
Marital status			3.33	0.068
Single	13 (18.31)	58 (81.69)		
Couple	84 (11.04)	677 (88.96)		
Residential region			0.22	0.640
Rural	55 (11.22)	435 (88.78)		
Urban	42 (12.28)	300 (87.72)		
Education, year			5.82	0.055
≤12	25 (8.50)	269 (91.50)		
13 ~ 15	44 (12.22)	316 (87.78)		
≥16	28 (15.73)	150 (84.34)		
Income			9.58	0.048
Q1 (Lowest)	14 (7.65)	169 (92.35)		
Q2	26 (17.11)	126 (82.89)		
Q3	16 (8.84)	165 (91.16)		
Q4	20 (14.29)	120 (85.71)		
Q5 (Highest)	21 (11.93)	155 (88.07)		
Professional title			0.408	0.815
None	36 (11.21)	285 (88.79)		
Primary	44 (12.46)	309 (87.54)		
Intermediate or higher	17 (10.76)	141 (89.24)		
Work experience, year			4.73	0.192
≤10	31 (15.20)	173 (84.80)		
11 ~ 20	28 (12.33)	199 (87.67)		
21 ~ 30	27 (8.97)	274 (91.03)		
≥31	11 (11.00)	89 (89.00)		
Chronic condition			11.45	0.001
Yes	31 (19.38)	129 (80.63)		
No	66 (9.82)	606 (90.18)		

^a^Chi-squared tests for differences of subclinical depression.

### 3.2. Association between occupational stress and subclinical depression

Table 3 shows the comparison of occupational stress between primary healthcare workers with and without subclinical depression. Overall, participants with subclinical depression had a significant higher level of occupational stress than those without subclinical depression (*p* < 0.001). When comparing the level of challenge stress and hindrance stress between the two groups, the group with subclinical depression also tended to reported higher levels of challenge stress and hindrance stress (both *p* < 0.001). Table 4 presents the associations of occupational stress and subclinical depression, which were estimated using multiple logistic regression models with unadjusted and adjusted covariates. As hypothesized, higher level of occupational stress was significantly associated with more severe subclinical depression, and the risk of subclinical depression remained after adjusting other covariates (OR = 4.57, 95%CI, 3.14–6.63). Notably, the association between challenge stress and subclinical depression was not statistically significant when controlling for hindrance stress (*p* > 0.05). Nevertheless, when adjusting for other covariates, both challenge-stress and hindrance-stress could significantly increase the OR of subclinical depression, and the OR (95%CI) was 1.52 (1.01–2.29) and 2.68 (1.98–3.69), respectively.

**Table 3:** Comparison of occupational stress by subclinical depression.

	Depression		*t*	*p*-value
	Yes	No		
Occupational stress (M ± SD)	3.79 ± 0.68	3.11 ± 0.69	−9.13	<0.001
Challenge stress (M ± SD)	4.18 ± 0.67	3.68 ± 0.74	−6.25	<0.001
Hindrance stress (M ± SD)	3.33 ± 0.87	2.43 ± 0.88	−9.45	<0.001

**Table 4:** Association between occupational stress and subclinical depression (*N* = 832).

	ORs (95%CI) of depression			
	Unadjusted model		Adjusted model^a^	
Occupational stress	4.12 (2.94, 5.79) ***		4.57 (3.14, 6.63) ***	
Challenge stress		1.43 (0.96, 2.14)		1.52 (1.01, 2.29) *
Hindrance stress		2.53 (1.88, 3.42) ***		2.68 (1.95, 3.69) ***

^a^Adjusted for age, sex, marital status, residential region, education, income, professional title, work experience, chronic condition; *p < 0.05; **p < 0.01; ***p < 0.001.

### 3.3. Subgroup analysis for sex and residential region groups

Table 5 demonstrates the results of subgroup analysis for sex and residential region groups. Pre-interaction tests showed that the association between occupational stress and subclinical depression differed by sex and residential region. As expected, after adjusting other covariates, the positive correlation between occupational stress and subclinical depression remained robust in the two subgroups, and was particularly stronger in male and rural groups (all *p* < 0.001). Meanwhile, the strong relationship between higher level of hindrance stress and the risk of subclinical depression was significant in all subgroup analyses (all *p* < 0.05). With respect to challenge stress, however, a slightly different pattern was shown between male and female. The regression model results indicated that the association between challenge stress and subclinical depression was not statistically significant in those female primary healthcare workers when controlling for hindrance stress and other covariates (*p* > 0.05). Besides, the similar results were found in both rural and urban primary healthcare workers, where the challenge stress had no significant effect on subclinical depression (all *p* > 0.05).

**Table 5:** Subgroup analysis of association between occupational stress and subclinical depression.

	*N* (%)	Adjusted ORs (95%CI) of depression	
Occupational stress × Sex^a^	832 (100.00)	1.22 (1.08, 1.39) **	
Occupational stress × Residential region^b^	832 (100.00)	1.38 (1.21, 1.56) ***	
Male^a^	444 (53.37)		
Occupational stress		5.56 (3.30, 9.36) ***	
Challenge stress			1.82 (1.02, 3.25) *
Hindrance stress			2.70 (1.79, 4.07) ***
Female^a^	388 (46.63)		
Occupational stress		3.29 (1.87, 5.80) ***	
Challenge stress			1.21 (0.66, 2.20)
Hindrance stress			2.59 (1.50, 4.46) **
Rural^b^	490 (58.89)		
Occupational stress		5.93 (3.47, 10.13) ***	
Challenge stress			1.60 (0.87, 2.95)
Hindrance stress			3.10 (1.98, 4.85) ***
Urban^b^	342 (41.11)		
Occupational stress		3.31 (1.92, 5.72) ***	
Challenge stress			1.45 (0.82, 2.57)
Hindrance stress			2.19 (1.34, 3.57) **

^a^Adjusted for age, marital status, residential region, education, income, professional title, work experience, chronic condition; ^b^Adjusted for age, sex, marital status, education, income, professional title, work experience, chronic condition; *p < 0.05; **p < 0.01; ***p < 0.001.

## 4. Discussion

This study explored the associations between occupational stress, including challenge stress and hindrance stress, and subclinical depression among Chinese primary healthcare workers. As expected, occupational stress, especially hindrance stress, has a significant impact on the occurrence of subclinical depression. Findings remained robust in subgroup analysis for sex and residential region groups after adjusting for other covariates.

Overall, the prevalence of subclinical depression among primary healthcare workers was 11.66% in the sample of Tai’an China. Previous studies have investigated the relationship between perceived stress and depression, and most of them are consistent with the findings of our study. However, the data of some recent studies were obtained during the peak of the COVID-19 pandemic, and healthcare workers on the frontlines were more susceptible to trauma and psychological deficits, which may lead to a higher prevalence of depression than this study. For example, in the Czech Republic, the prevalence of depression increased from 11% after the first wave of the COVID-19 pandemic (June to August 2020) to 22% at the peak of the second wave of the pandemic (February to April 2021) (20). Our study was conducted in August, 2020, when China had transitioned to the mitigation phase after the first wave, and the depressive symptoms and stress of medical staff may have been relieved. Meanwhile, some studies conducted in China during the peak of the first wave (from January to March 2020) also showed a higher prevalence of depression. In a cross-sectional study of 1,090 medical staff in China, from February to March 2020, the estimated self-reported rate of depression symptoms was 18.4% (21). Another study indicated that 50.4% of respondents reported depression symptoms among 1,257 health care workers in 34 hospitals equipped with fever clinics or wards for patients with COVID-19 (22). In addition, the prevalence of depression may vary widely due to different settings and applied instruments in each study (23). For example, Ahmed et al. found that up to 60% of healthcare workers on the frontlines in Egypt and Saudi Arabia experienced depression [measured by Depression Anxiety Stress Scale-21 (12)]. Another possible explanation for the lower prevalence of depression in our study was that the National Health Commission in China stipulates that primary health institutions do not undertake the task of treating patients with COVID-19, and mainly focus on monitoring, quarantine, report and referral of patients or suspected patients. The World Health Organization has announced that COVID-19 is no longer a public health emergency of international concern, and China has transited from emergency mode to managing COVID-19 alongside other infectious diseases. However, China still faces the risk of epidemic spreading due to not only to sporadic new cases and asymptomatic carriers but also ever-mutating coronavirus, and primary medical staff always bear heavy work and are worried about being infected, and their subclinical depression still warrant attention.

The main results of this study also indicated that primary healthcare workers who perceived a higher level of occupational stress, especially for hindrance stress, tended to develop subclinical depression symptoms. Similar to findings from other study, a high level of stress was associated with a high degree of emotional exhaustion as well as lack of personal accomplishment among physicians in primary health care (24). Meanwhile, higher level of perceived stress was significantly positively related to severe insomnia and depression among medical staff (25). As expected, these results manifest that the increased level of stress led to poorer depression symptoms, however, further more studies are required to determine whether this is a long-term effect. Moreover, previous studies have indicated that despite being the backbone of the primary healthcare workforce, primary healthcare doctors in China were considerably underpaid, often without legally mandated social benefits, and are commonly exhausted (26, 27). The high levels of challenge and hindrance stress among primary healthcare workers would necessitate effective coping strategies. Evidence has shown that appropriate level of challenge stress was recommended among young workers, but at the same time, interventions targeting hindrance stress should be developed and implemented for healthcare workers to raise job performance and improve quality of healthcare (28, 29). Psychosocial interventions focused on cognitive and behavioral principles proved the strongest evidence, particularly for reducing occupational stress among medical staff (2). Therefore, in addition to expanding the total amount of primary healthcare human resources, more attention should be paid to the establishment of a scientific and reasonable incentive mechanism, including increasing material and non-material motivations to alleviate the hindrance stress of primary medical staff.

Furthermore, this study showed subtle sex differences for the association between challenge stress and subclinical depression, that is, those male healthcare workers who perceived higher level of challenge stress were more likely to develop subclinical depression. This may be due to the fact that female healthcare workers reported lower level of occupational stress than male in this study, and they may have a more conservative threshold for the perception or reporting of stressors. Our finding was also consistent with a previous study from American that suggested that stressful life events were a stronger predictor of subsequent clinical depression in men compared to women (30). Despite being in the same occupational category, there may be differences in work tasks between men and women, which may contribute to the observed differences to a certain extent. In China primary health facilities, male healthcare workers usually take on lager workloads and cope with more complex medical problems, which may increase their feelings of irritation and anxiety (31). Accordingly, more targeted and appropriate interventions should be conducted, especially for male healthcare workers, to decrease the detrimental challenge stress effects associated with subclinical depression.

This present study adds to the increasing literature regarding the association between occupational stress and subclinical depression among primary healthcare workers in China. However, the limitations of this study should also be noted. First, the cross-sectional design restricted our ability to determine the cause and effect between occupational stress and subclinical depression, therefore, more longitudinal studies are warranted. Second, there may be more confounding factors than those available for consideration in this study. Finally, this study was conducted in a single city, which may limit the generalization of the results across the country.

## 5. Conclusion

In summary, the prevalence of subclinical depression among primary healthcare workers was high. Occupational stress, especially hindrance stress, was significantly positively associated with subclinical depression among primary healthcare workers in China. Considering the impact of high level of hindrance stress on increasing the occurrence of subclinical depression among primary healthcare workers, improving work condition and minimizing hindrance stress in the workplace should be a priority in the primary healthcare setting. Furthermore, findings from our study are important for identifying population subgroups in which primary healthcare providers’ psychological intervention programs are potentially most cost-effective.

## Data availability statement

The raw data supporting the conclusions of this article will be made available by the authors, without undue reservation.

## Ethics statement

The studies involving humans were approved by the Ethical Committee of Shandong University School of Public Health. The studies were conducted in accordance with the local legislation and institutional requirements. The participants provided their written informed consent to participate in this study.

## Author contributions

JZ and LX conceived and designed the study. JZ conducted the analysis and wrote the initial draft. WQ and AX contributed to the interpretation of the findings and made critical revisions to the manuscript. All the authors approved the version to be published.
